# End-of-life decision-making in very old critically ill patients

**DOI:** 10.1007/s41999-026-01518-7

**Published:** 2026-06-12

**Authors:** Sigal Sviri, Isao Nagata, Wojciech Szczeklik, Charles L. Sprung

**Affiliations:** 1 Department of Medical Intensive Care, Hadassah Medical Center and Faculty of Medicine, Hebrew University of Jerusalem, Ein Karem, Israel; 2 Department of Intensive Care Medicine, Yokohama City Minato Red Cross Hospital, Yokohama, Japan; 3 Health Services Research and Development Center, University of Tsukuba, Tsukuba, Japan; 4 Centre for Intensive Care and Perioperative Medicine, Jagiellonian University Medical College, Krakow, Poland; 5 Department of Anesthesiology, Critical Care and Pain Medicine, Hadassah Medical Organization and Faculty of Medicine, Hebrew University of Jerusalem, Jerusalem, Israel

**Keywords:** End-of-life, Very old, Shared-decision-making, Withholding, Withdrawing, Palliation

## Abstract

End-of-life decision-making in the intensive care unit (ICU) is ethically complex, particularly for very old patients with limited physiological reserve. Decisions to limit life-sustaining treatment are shaped by medical prognosis, patient values, cultural and socioeconomic contexts, and uncertainty regarding outcomes. Non-beneficial treatment refers to interventions unlikely to provide meaningful benefit while imposing additional burden or suffering, and avoiding such treatment is central to high-quality end-of-life care. Limitation of life-sustaining treatment may involve withholding or withdrawing therapies, which are distinct from euthanasia, and should be accompanied by a transition toward comfort-focused care. Frailty, pre-illness functional status, and illness severity are key determinants of outcomes and treatment decisions in very old ICU patients. Large international studies, including the VIP, VIP2, and COVIP cohorts, demonstrate that frailty is strongly associated with mortality, functional decline, and treatment limitation, while revealing substantial regional variation in end-of-life practices. Prognostication remains imprecise, often leading to prolonged aggressive care despite poor long-term outcomes. Shared decision-making, grounded in transparent communication and alignment with patient goals, is the ethical standard but is frequently undermined by discordance between documented preferences and delivered care. When decision-making capacity is lost, in the majority of countries shared decision-making extends to surrogate decision-makers, who often experience emotional burden and limited accuracy in predicting patient preferences. Ongoing, structured conversations, meticulous documentation, and time-limited trials of intensive therapy can support iterative reassessment of goals of care.

## Key summary points

**Aim:**

We examine the ethical, clinical, and practical challenges of end-of-life decision-making in very old critically ill patients.

**Findings:**

Frailty, baseline functional status and severity of acute illness are major determinants of outcomes and treatment decisions. As prognostic uncertainty remains substantial, time-limited ICU trials, transparent communication and shared decision-making may improve goal-concordant care and reduce non-beneficial treatment.

**Message:**

End-of-life care in very old ICU patients is challenging and should prioritize patient values, dignity, comfort,and quality of life, while integrating clinical expertise with careful, supportive decision-making processes.

## Introduction

Population ageing is reshaping intensive care practice worldwide [1]. Patients aged 80 years and older represent a rapidly growing proportion of intensive care admissions and are characterized by marked heterogeneity in baseline health, physiological reserve, and recovery potential. As a result, decisions about escalation, continuation, or limitation of life-sustaining treatments (LST) are frequent and often occur under conditions of prognostic uncertainty [2].

In very old critically ill patients, the clinical question is commonly not whether intensive therapies can be delivered, but whether they are likely to achieve outcomes that the patient would consider acceptable. Frailty, pre-illness function, comorbidity, and acute illness severity inform these judgments, yet no single measure can fully capture an individual patient’s trajectory [3]. Decision-making therefore relies on the clinician assessment of prognosis, followed by a shared decision-making process, which integrates the best available evidence with the patient’s values, goals, and preferences, and supporting surrogate decision-makers [4, 5].

This review summarizes key concepts and practical considerations in end-of-life decision-making for very old ICU patients. We address definitions of non-beneficial treatment, prognostic uncertainty and the role of baseline vulnerability; patient values and surrogate decision-making; communication strategies and approaches to withholding and withdrawing LST [6]. We discuss the importance of limiting suffering and promoting palliation and discuss their implications, including stress and burden on family members, conflict, moral distress, and burnout of clinicians. We conclude with future directions for education, research, and system-level support to promote goal-concordant, patient- and family-centered care.

## Methods

This review was written by established experts in the field. Each section was written after extracting the most valuable papers dealing with end-of-life issues, focusing on very old critically ill patients.

### Definition of and indications for end-of-life decisions

End-of-life (EOL) decision-making in the ICU is a complex and ethically challenging process [4]. The terms “non-beneficial” or “inappropriate” treatment have been proposed instead of the term futile therapy in the context of end-of-life care [7–9]. Furthermore, indications for limiting LST vary across geographical regions, cultures, religious traditions [10], and even economic contexts, including differences related to national health expenditure or gross domestic product (GDP). Importantly, decisions about treatment limitation are often particularly difficult in very old patients, in whom the balance between prolonging life and preserving dignity, comfort, and acceptable quality of life becomes especially relevant [6].

To support clinicians in these situations, several professional bodies have developed recommendations and consensus statements on end-of-life care in critically ill patients [4]. These widely cited frameworks emphasise transparent communication, ethical decision-making, and avoidance of non-beneficial treatments, ensuring that medical interventions remain aligned with the patient’s best interests and goals of care [11, 12].

Non-beneficial treatment can be defined as a medical intervention that, according to current scientific knowledge and expert clinical judgement, is unlikely to provide meaningful clinical benefit while exposing the patient to additional harm, burdens, or adverse effects [8, 13]. In the context of terminal illness, non-beneficial treatment may involve continuation or initiation of life-sustaining interventions aimed solely at maintaining physiological functions in patients with no realistic prospect of recovery [9]. Such interventions may prolong the dying process and can be associated with additional suffering and/or violation of the patient’s dignity. Avoiding non-beneficial treatment therefore represents an essential component of high-quality end-of-life care [8, 14].

Two principal approaches are used in clinical practice to avoid non-beneficial therapy through limitation of life-sustaining treatment: withholding and withdrawing interventions [6]. Withholding treatment refers to the decision not to initiate a new life-sustaining intervention or not to escalate existing therapy, whereas withdrawing treatment involves discontinuing a therapy that has already been started, such as mechanical ventilation, vasopressor support, or renal replacement therapy. Although withdrawal of treatment may appear ethically more challenging, most contemporary ethical frameworks consider withholding and withdrawing therapies to be morally equivalent, as both aim to serve the patient’s best interests and avoid unnecessary suffering [15]. Importantly, the limitation of non-beneficial treatment should not be confused with euthanasia. Instead, it represents a transition from curative or life-prolonging intent toward comfort-focused care, accompanied by the establishment of appropriate treatment limitations and the prioritization of symptom control, dignity, and quality of life [16, 17].

Several clinical triggers may prompt clinicians to consider the possibility of treatment limitation in very old critically ill patients. These include persistent organ failure despite maximal therapy, severe frailty or advanced cognitive impairment, survival associated with a quality of life that may be considered unacceptable, as well as prolonged mechanical ventilation or repeated ICU admissions [6, 18]. Frailty, in particular, has emerged as an important prognostic factor in older patients and can be assessed using validated tools such as the Clinical Frailty Scale, which provides a global measure of physiological reserve and vulnerability in elderly individuals.[19]. Frailty has also been consistently associated with increased mortality and poorer functional outcomes in critically ill older adults.[20].

Important insights into end-of-life decision-making in very old ICU patients were provided by the VIP (Very Old Intensive Care Patients) study group [21]. In a large prospective cohort study including 5021 patients aged ≥ 80 years admitted to 309 ICUs across 21 European countries, limitation of LST occurred in 27.2% of patients, including 15% with withholding decisions and 12.2% with withdrawing decisions [22]. Patients with LST limitation were generally older, more frail, and more severely ill, and were less often admitted electively. ICU and 30-day mortality were 29.1% and 53.1% in the withholding group and 82.2% and 93.1% in the withdrawing group, respectively. The study also revealed substantial regional variation: treatment limitation was less frequent in Southern and Eastern Europe and more common in Northern Europe.

Large international observational studies of older ICU patients, including the VIP2 study and the COVIP study, have further demonstrated that frailty, acute illness severity, and pre-existing functional status strongly influence survival and treatment decisions in this population, reinforcing the need for careful assessment of baseline vulnerability when considering treatment limitation [23, 24].

In situations where prognosis remains uncertain, clinicians may consider implementing a time-limited trial of intensive therapy [25]. This approach allows the healthcare team and family to evaluate the patient’s response to treatment over a predefined period before reassessing goals of care and determining whether continuation of life-sustaining therapy remains appropriate [26].

### Prognostic uncertainty and the central role of pre-illness function

Decisions to admit or refuse admission of critically ill patients to the ICU are difficult, especially for the elderly. The biological and functional heterogeneity in very old patients constitutes a major challenge to prognostication and ICU patient management [2, 27, 28]. In addition to different acute diseases, frailty, comorbidities, cognitive impairment, and functional disabilities influence outcome in the elderly [18, 23]. Repeated reassessments over time are more reliable than single-point predictions.

Pre-ICU status is extremely important. A prospective study of 242 ICUs in 22 countries confirmed that frailty using the Clinical Frailty Scale predicted one month mortality in elderly patients 80 years or above admitted to the ICU [23]. Among older persons with critical illness, more than half died within 1 month or experienced significant functional decline over the following year, with particularly poor outcomes in those who had high levels of premorbid disability [29].

Doctor prognoses for terminally ill patients are inaccurate and overly optimistic [30] leading to continued aggressive care [31]. Current approaches for prognosticating outcomes in elderly patients are inadequate and introduce uncertainty about the best trajectories for ICU care in these individuals [2, 25]. Statistical models developed for predicting the probability of ICU survival in large patient cohorts do not work well in very old patients. This is due to methodological problems related to the heterogeneity of the elderly along with the differences in cultural attitudes, available resources, disparities in legal and professional norms, especially in patients with multiple morbidities and underlying impairments [32]. In a multicenter study of 242 ICUs in 22 countries of patients aged 80 years old or older without limitations of life-sustaining treatment, ICU mortality was 12% and 30-day mortality 19%. Phenotypes with distinct profiles of high disease severity and geriatric characteristics were identified in these patients with a 50% ICU mortality and 57% 30-day mortality [27].

Family members of patients aged 80 years or older admitted to 22 ICUs reported that their most important value in making treatment decisions was the "patient be comfortable and suffer as little as possible" and their least important value was "the belief that life should be preserved at all costs" [33]. Overall, 29.7% of patients received life-prolonging therapies for greater than 7 days and 50.3% of them died in the hospital. Deficiencies in communication and decision-making may be associated with prolonged use of life-sustaining treatments in elderly critically ill patients, many of whom ultimately die [31, 33].

In a multicenter study of 24 Canadian ICUs in patients 80 years or older one third of the patients died in the hospital, many after a prolonged ICU stay while continuing to receive aggressive life-sustaining interventions. Unfortunately, the presence of frailty or advance directives had little impact on limiting use of life-sustaining treatments or shortening the time from admission to death [31].

An observational study of triage decisions in eleven ICUs in seven European countries over eighteen months demonstrated that ICU refusals and mortality rates increased with increasing patient age [34]. Controlling for underlying or acute disease, age and not underlying or acute diseases accounted for increased mortality. Mortality differences between accepted and rejected patients, however, were greatest for older patients greater than 65 years [34]. When the reason for ICU rejection was “too ill,” mortality was very high in the young and old but when the reason for rejection was “too well,” mortality was much greater in patients greater than 65. The elderly patient triaged for ICU admission who is “too well” responding to fluids for hypotension or responding with improved saturations to oxygen therapy is at high risk for mortality and should be admitted to an ICU.

Because of the prognostic uncertainty, health care professionals must ascertain the preferences of patients who may prioritize functional outcomes over survival. [26]. Time-limited ICU trials may be an appropriate approach to increase confidence in decisions to limit life-prolonging therapies [25], improve clinician/family meetings and decrease the intensity and duration of ICU interventions [35].

Although time-limited trials are used to decrease prognostic uncertainty in patients by evaluating the response to therapy over time, it is unclear how long the trial should be [25]. To address this question in patients aged 80 years or older, periods of increased prognostic uncertainty were identified during the first 7 days after admission. The duration of uncertainty depended on baseline patient characteristics including frailty, illness severity, and organ support [26].

The goal of ICU care is not only to avoid death, but more importantly to maintain an acceptable quality of life and functional autonomy after hospital discharge [36].

### Patient values, preferences, and surrogate decision-making

A multinational survey study reported that the patient's values are one of the key parameters in decision-making for very old ICU patients [32]. Regarding the values of old critically Ill patients, they desire not merely survival, but the avoidance of cognitive or functional impairment. A multicenter questionnaire survey showed that most seriously ill patients would not choose treatment if the outcome was survival accompanied by severe functional or cognitive impairment [12]. In addition, a prospective cohort study reported that a majority of seriously ill patients considered bowel and bladder incontinence, requiring a breathing tube or a feeding tube to live, and needing care from others, the same or worse than death [37]. Despite these patient values, there is frequent discordance between patient preferences and the actual medical setting. A controlled trial showed substantial discordance between patients' preferences for life-sustaining treatment and clinicians' perceptions and documentation in medical records [38]. A multicenter prospective study reported that agreement between patients' expressed preferences for end-of-life care and orders of care documented in the medical record was only 30.2% [39]. Furthermore, a multicenter prospective observational study reported disagreement between clinicians and patients or their surrogates regarding the appropriateness of treatment in approximately one-third of ICU patients [40].

When patients’ decision-making capacity is lost, patient-designated and next-of-kin surrogates make end-of-life treatment decisions on their behalf; unfortunately, the accuracy of surrogate predictions regarding patients' treatment preferences are limited. A systematic review reported that patient-designated and next-of-kin surrogates incorrectly predict patients' end-of-life treatment preferences in one third of cases [41]. Beyond this predictive inaccuracy, surrogate decision-making imposes a substantial emotional burden [42]. A systematic review showed that at least one-third of surrogates experienced negative emotional burdens—including stress, guilt over the decisions they made, and doubt regarding whether they had made the right decisions—which were often substantial and typically lasted months or sometimes years as the result of making treatment decisions [43]. A prospective observational study reported that post-traumatic stress reactions consistent with a high risk of Post-Traumatic Stress Disorder (PTSD) were common in family members of ICU patients and were especially prevalent among those involved in end-of-life decision-making [44]. Decreased functional status and cognitive decline after ICU discharge were identified as the most significant contributors to caregiver burden. [45].

According to an American College of Critical Care Medicine and American Thoracic Society Policy Statement, when patients lack decision-making capacity, surrogates use substituted judgment when the patient’s preferences are known; when they are not known, the best interest standard guides end-of-life decisions [46]. However, several challenges remain for surrogates and clinicians in understanding patients’ values and preferences, as well as for surrogates and clinicians in making end-of-life treatment decisions [47]. To address these problems, advance care planning, transparent communication of functional prognoses, and supportive frameworks that recognize both the ethical complexity and psychological weight of surrogate decision-making are required.[48]

### Shared decision-making and communication in the ICU

Shared decision-making (SDM) has become the ethical and professional standard for treatment decisions in the intensive care unit. According to an American College of Critical Care Medicine and American Thoracic Society Policy Statement, SDM was defined as a collaborative process that allows patients, or their surrogates, and clinicians to make health care decisions together, taking into account the best scientific evidence available, as well as the patient's values, goals, and preferences [46]. Therefore, SDM is not merely the disclosure of medical information or the passive presentation of therapeutic options. Effective SDM requires clear recommendations from clinicians that are consistent with the patient’s values. In addition, decision-making should be focused on goals of care rather than isolated procedures [47]. Thus, rather than concentrating solely on whether to initiate or withhold specific interventions such as dialysis or prolonged mechanical ventilation, clinicians are encouraged to clarify the overarching goals of care.

Goals of care should be decided based on patient’s values and preferences. These goals may be addressed through fundamental questions such as: What outcomes are most important to the patient? Which health states would the patient consider acceptable, and which would be intolerable? Equally important is the exploration of treatment burdens, including prolonged dependence on mechanical devices, severe cognitive impairment, or loss of functional independence [46]. By clarifying acceptable and unacceptable burdens, clinicians and surrogates can consider treatments according to whether they support or undermine the patient’s life priorities. A descriptive qualitative study reported that relationship, place, independence, and physical comfort were the most frequently documented goals of care among adult ICU patients with life-limiting illnesses, whereas longevity was the least frequently identified [47]. In addition, goals-of-care recognize that patient preferences for LST are dynamic rather than fixed. A multicenter, prospective, longitudinal study demonstrated that more than one-third of outpatients with advanced chronic obstructive pulmonary disease, congestive heart failure, or chronic renal failure changed their preferences regarding cardiopulmonary resuscitation and/or mechanical ventilation at least once during a one-year period [49]. Consequently, SDM in the ICU should not be viewed as a single decisional event but rather as an iterative process requiring ongoing reassessment as clinical circumstances evolve, particularly when patients experience change in health status, mobility, symptoms of anxiety or depression, or marital status.

To implement SDM, structured family meetings, repeated clarifications, avoidance of “choice overload” and meticulous documentation are essential. Structured family meetings help establish a partnership among clinicians, patients, and surrogates. Clinical practice guidelines from the American College of Critical Care Medicine recommend that family meetings with the multiprofessional ICU team begin within 24–48 h of ICU admission and be scheduled at regular intervals and as needed [42]. In addition, clinical trials reported that conducting family conferences within 72 h of ICU admission was associated with fewer ICU days among patients who subsequently die and with higher ratings of the quality of dying reported by family members [50, 51]. Repeated clarification is also important because patients and surrogates may have varying levels of comprehension for patients’ medical condition and their preferences for LST may change dynamically over time [52]. At the same time, clinicians must avoid the risk of “choice overload.” In SDM, treatment recommendations should reflect the patient’s values, goals, and preferences while also incorporating the clinician’s professional judgment regarding which options are medically appropriate [46]. Therefore, clinicians should present options that are aligned with the patient’s values and goals while guiding decision-making through their clinical expertise. Finally, meticulous documentation is crucial for SDM. Clear documentation identifying surrogate, summarizing patient values, articulating established goals of care, and specifying treatment limitations promote continuity of treatment, ensures consistency among clinicians, patients, and surrogates, and supports ethical transparency [53].

### Limitation of treatment: DNR, withholding, and withdrawing therapies

ICUs around the world treat the most critically ill patients and save lives every day. Unfortunately, some patients are unlikely to survive and decisions are made to withhold or withdraw their LST. Withholding treatment is defined as a decision not to start or increase a LST and withdrawing treatment is a decision to actively stop a LST presently being given [6, 54]. The commonly withheld or withdrawn LSTs include cardiopulmonary resuscitation (CPR), mechanical ventilation, and vasopressors and renal replacement therapy (1). Studies demonstrate that there is more withholding than withdrawing LSTs (1).

Country variability in end-of-life practices between countries is related to several factors including physician, patient, and institutional characteristics, culture, geography, economics, laws, religion, religiosity, and ethics education [10, 55]. Over time, limitations in LSTs have occurred more frequently and death without limitations occurred less frequently [54]. Interestingly, there were higher survival rates after limitations of LSTs so decisions to limit LSTs do not necessarily mean the patient will die.

In a multicenter study of 5021 patients aged ≥ 80 years in 309 ICUs from 21 European countries, 72.8% of patients had no LST limitations, 15.0% had withholding LST, and 12.2% withdrawing [6]. Patients with LST limitations were older, more frail, more severely ill, and were electively admitted less. The ICU and 30-day mortality were, respectively, 29.1 and 53.1% in the withholding group and 82.2% and 93.1% in the withdrawal group [6]. End-of-life limitations were highest in patients ≥ 80 years old (88.2%) and lowest in < 65 years old (82.5%), with withholding therapy highest in the ≥ 80 years group (56.1%) and withdrawing therapy highest in the < 65 years group (41.1%) [56]. In a study looking at the differences in end-of-life practices in 3105 critically ill COVID-19 patients aged 70 years and older across Europe, it was found that northern countries had a higher rate of end-of-life practices but with a similar mortality rate [57].

The majority of intensivists, ethicists, and medical organizations state that there is no moral difference between withdrawing life-sustaining treatments and withholding them [15]. Despite this fact physicians are more disturbed at withdrawing than withholding therapy and the General Medical Council guideline states “it may be emotionally more difficult … to withdraw a treatment … than to decide not to provide a treatment in the first place” [58]. Most experts disagree (77%) that age should be used as the sole criterion for withholding or withdrawing LST, and 91% disagree that there should be a specific age for these decisions [11]. Nevertheless, 91% acknowledge that age should be a significant consideration with other factors.

As the rapid withdrawal of oxygen or mechanical ventilation may lead to dyspnea, the gradual reduction of ventilatory support may allow time to control dyspnea; alternatively recommendations for more rapid withdrawal of ventilatory support has included greater titration of medications [4]. Withdrawal of ventilatory support and other LSTs should occur with proactive symptom management for dyspnea, pain, and agitation and proportionate sedation ensuring the comfort of the patient through the dying process [4].

Limitations of LST often occur to respect the autonomy and the wishes of the patient, avoid prolonged suffering, or escape a poor quality of life. Regrettably, “do not resuscitate” (DNR) orders occasionally become “do not care” expressions with the avoidance of patients who require more care. To prevent this, targeted education and training programs for students and medical staff caring for these patients must be implemented [59]. In addition, the use of “slow codes” instead of performing CPR is inappropriate [60].

### Conflict, moral distress, and institutional support

Conflicts arise when families request non-beneficial life-prolonging treatments or when religious or cultural views differ from the physician’s clinical judgment. ICU interventions are considered inappropriate when there is no reasonable expectation the patient will survive or no expectation that neurologic function will improve for the patient to perceive treatment benefits [8]. The phrasing "potentially inappropriate", rather than futile, is preferred to depict treatments that may attain the outcome the patient desires, but doctors believe opposing facts warrant not administering them [9]. The term "futile" should be confined to when requested interventions cannot accomplish their intended physiologic goal [9].

Conflicts undermine patient safety, patient/family-centered care, and staff welfare [61]. They generate staff burnout and increase healthcare costs [61]. Conflicts about potentially inappropriate treatments remaining after intensive communication and negotiation should be handled by conflict resolution including hospital review, finding another provider at another institution, and external review of decisions [9]. When it is impossible to carry out the conflict-resolution process and doctors believe the requested treatment is outside accepted practice, oversight should be obtained before avoiding the provision of the requested treatment.

In a study of 7,498 ICU staff members from 323 ICUs in 24 countries, over 70% of ICU staff disclosed conflicts, 53% were severely associated with job strain [62]. Nurse-physician conflicts were the most common (32.6%), then nurse conflicts (27.3%), with 26.6% staff-relative conflicts. Conflicts stemmed from increased workload, inadequate communication, and end-of-life care [62] and can lead to moral distress and burnout. Conflict prevention requires improved understanding of family experiences, preferences, and values [61]. Ethics consultations, clear institutional frameworks, and transparent communication often reduce conflict and support both families and staff.

Contributing factors to moral distress in the ICU are the organizational climate and the ICU clinical climate with themes related to powerlessness, end-of-life care, ineffective teamwork, and personal characteristics [63]. Situations with higher levels of moral distress include inappropriate care that prolongs death, unnecessary treatments and tests, working with incompetent healthcare personnel [64], less experience working in ICUs, being younger, female, and intending to leave employment [65], and the "provision of less-than-optimal care" and "caring for patients they did not feel qualified to care for" [66]. Strategies for reducing moral distress include the use of self-adjustment procedures, institutional support enhancing staffs' moral resilience, advancing reflective thinking and improving communication [67].

Nurse burnout is prevalent worldwide, manifested by a feeling of inferior personal achievement [68]. Burnout threatens patient care quality, safety, and outcomes, concurrently harming nurses' mental health, job satisfaction, and retention [68]. It also imposes financial burdens on healthcare institutions with absenteeism, decreased productivity, and higher turnover. Factors contributing to burnout include role conflict, negative emotions, family issues, moral distress, stress, distance from work, predictability of work tasks, and institutional advancement. A French national survey of 189 ICUs found approximately one-half of intensivists had a high level of burnout [69]. Female sex and workload but not aspects related to patients were associated with burnout.

Institutional policies can help support staff and reduce burnout and moral distress [35, 70]. Approaches to increase resilience and well-being include self-awareness, self-care, situational awareness, and training to increase confidence and abilities for providing end-of-life care [71]. Reactive strategies include case conferences, pre-briefings and de-briefings.

### Transition to comfort-focused care and palliative integration

Palliative care aims to improve quality of life through early identification, prevention, and relief of suffering, and can be provided throughout the course of serious illness [17]. Patients and families facing critical illness have palliative care needs, regardless of patient’s diagnosis or prognosis, and palliative care is often optimally provided together with life-sustaining treatment. Within this continuum, comfort-focused care represents an approach that prioritizes relief of distressing symptoms, such as pain, dyspnea, agitation, and delirium, particularly when the primary goal of care shifts toward comfort, although elements of this approach may be applied alongside life-prolonging therapy. Therefore, palliative care is not a mutually exclusive alternative, nor simply a sequel to failed attempts at life-sustaining treatment, but rather an integral component of comprehensive care for critically ill patients from the time of ICU admission [72]. In addition, transitioning to comfort care, which focuses on symptom control and is typically provided in the final phase of life [17], should be framed as a care goal-consistent shift.

In comfort-focused care, effective symptom control is a central goal of care. Common symptoms include dyspnea, pain, agitation, and terminal delirium. The cornerstone of effective symptom control is systematic symptom assessment with tools, and pharmacologic and nonpharmacologic interventions are used to relieve these symptoms [73, 74]. Through these interventions, critically ill patients may experience reduced suffering and be allowed to experience the dying process with as much comfort and dignity as possible.

Collaboration with palliative care specialists is another essential component of comfort-focused care in the ICU. In the most common model, ICU clinicians serve as the primary providers of palliative care, with specialist consultation for more complex situations [75, 76]. The effectiveness of this model depends on close interprofessional collaboration between ICU and palliative care teams in supporting patients and families. Fragmented or poorly integrated specialist involvement may be ineffective or even harmful, underscoring that meaningful integration requires true collaboration rather than episodic consultation [77].

Furthermore, supporting families during the dying process is an important component of comfort-focused care. Family members of critically ill patients frequently experience substantial emotional distress, including anxiety, depression and grief. To support such family members, communication and family-centered care are necessary; written informational materials (e.g., brochures or leaflets), communication by ICU clinicians trained in palliative care and structured approach for conducting end-of-life family conference [75].

Ultimately, integrating palliative care and facilitating a timely transition to comfort-focused care enables ICU clinicians to align treatments with patient goals while minimizing patients' unnecessary suffering at the end-of-life.

## Limitations

This review summarizes key concepts and practical considerations in end-of-life decision-making for old ICU patients. We address definitions of non-beneficial treatment, prognostic uncertainty and the role of baseline vulnerability; patient values and surrogate decision-making; communication strategies and approaches to withholding and withdrawing LST. We could not deal with the many issues confronting physicians in this patient population including low-middle income countries, changes of ethical principles during a surge period, or distributive justice. We did, however, include information related to some key issues including the COVID-19 pandemic and ICU triage of the elderly.

## Conclusions and recommendations

The ageing population and its needs at the end-of-life are challenging [78]—see Table 1. Mostly challenging is our need to focus on patient care and preferences, taking into consideration the ethical, moral, legal and social constraints on system implementation of end-of-life decisions [5]. Future challenges include developing and implementing curricula for students and ICU staff on shared decision-making, culturally sensitive communication, advanced care planning, and documentation of goals of care, including simulation-based training for difficult conversations [61]. Palliative care skills among ICU clinicians should be strengthened and collaboration with geriatricians and palliative care specialists should be encouraged [1, 36, 79]. Goals of care need to be better defined by emphasizing quality of life, limiting family stress, burden and team burnout. Social, ethical and moral conflicts can be reduced by better understanding approaches to end-of-life care across different ethnic and religious communities, as well as at the national and international level [4]. Further research should be targeted at advancing our knowledge and level of care in treating very old critically ill patients during their acute illness and at the end-of-life.

**Table 1:** Challenges in end-of-life decision-making in very old critically ill patients

Challenge	Description	Possible solutions/strategies
Prognostic uncertainty	Difficulty predicting outcomes due to heterogeneity and non-validated scores	Repeated assessments; frailty tools; time-limited trials; improved prognostic models
Heterogeneity of elderly patients	Wide variability in baseline health and recovery potential	Individualized assessment; avoid age as sole criterion
Discordance between preferences and care	Low agreement between documented preferences and delivered care	Advance care planning; structured goals-of-care discussions
Inaccurate surrogate decision-making	Surrogates incorrectly predict preferences in ~ 1/3 cases	Early discussions; advanced directives; structured communication
Emotional burden on surrogates	Stress, guilt, PTSD among family decision-makers	Psychological support; structured family meetings; palliative care
Communication failures	Delayed/poor communication leading to prolonged aggressive care	Early and repeated meetings; trained communication; avoid overload
Cultural and socioeconomic variability	Regional differences in end-of-life practices	Culturally sensitive frameworks; adapt guidelines
Withholding vs withdrawing difficulty	Withdrawal perceived as more difficult emotionally and morally	Education; structured protocols; team support
Overuse of life-sustaining treatments	Prolonged aggressive care despite poor outcomes	Time-limited ICU trials; reassessment; palliative integration
Conflict between families and clinicians	Frequent disagreements impacting care	Conflict resolution; ethics committees; transparent communication
Moral distress among clinicians	Perceived inappropriate care and powerlessness	Institutional support; debriefings; resilience training
Burnout among ICU staff	High prevalence affecting care quality	Improve work conditions; support systems; staffing
Changing patient preferences	Preferences evolve over time	Continuous reassessment; iterative shared decision making
Poor documentation	Leads to inconsistency across providers	Standardized documentation tools
Family burden post-ICU	Long-term psychological burden	Family-centered care; follow-up programs
System-level limitations	Lack of training and frameworks	Develop curricula; simulation training; policy development
